# Duration of sick leave after operated and non-operated distal radial fracture: a Finnish cohort study of 19,995 patients

**DOI:** 10.1177/17531934231194673

**Published:** 2023-08-25

**Authors:** Maarit Ax, Vili Palola, Ville Ponkilainen, Antti P. Launonen, Ville M. Mattila

**Affiliations:** 1 Faculty of Medicine and Health Sciences, University of Tampere, Tampere, Finland; 2 Department of Surgery, Central Finland Central Hospital Nova, Jyväskylä, Finland; 3 Department of Orthopedics and Traumatology, Tampere University Hospital, Elämänaukio, Tampere, Finland

**Keywords:** Distal radius fracture, operative treatment, non-operative treatment, absence from work, absenteeism, return to work

## Abstract

The purpose of this study was to investigate whether operative treatment for distal radial fracture reduces the length of sick leave and the costs of treatment. We identified 19,995 patients from a registry who received a state sick leave allowance between 2010 and 2019 owing to distal radial fractures. We compared these patients to a registry of operations and identified 4346 operated patients. Operated patients had a mean sick leave of 75 days, whereas non-operated patients had a sick leave of 63 days. In the operated group, the cost of sick leave was €7505 (UK£6419; US$8070), which was 34% higher than in the non-operated group. Over the analysed period, the duration of sick leave decreased. Although several studies have shown better early functional outcomes after operation, this does not seem to shorten sick leave.

**Level of evidence:** III

## Introduction

Although a distal radial fracture (DRF) is the most common injury among adults (Court-Brown and Caesar, 2006; Flinkkilä et al., 2011; Ponkilainen et al., 2022), the optimal treatment for DRF has been a matter of debate for decades. At present, the most common treatment for DRF is immobilization with a cast (Solvang et al., 2018). However, the incidence of operative treatment is increasing (Mattila et al., 2011; Mellstrand-Navarro et al., 2014). The surgical treatment of DRF has shifted towards treatment with open reduction and internal fixation, owing to the rapid and major development of anterior locking plates in the 1990 s, and the number of fixations with K-wires and external fixations have decreased during the same period in the Nordic countries (Hevonkorpi et al., 2018; Palola et al., 2021). Operative treatment has been shown to have advantages and seems to result in better functional results in the short term (Linnanmäki et al., 2023). It has also been suggested that it reduces costs of treatment (Mulders et al., 2020).

Because DRF is a common injury, it results in major costs for societies globally. These include not only the costs of treatment, but also the costs of rehabilitation, absence from work (sick leave) and functional disability (Lyons et al., 2011). In a recent meta-analysis, the mean duration of sick leave after DRF was estimated to be 86 days, varying between 68 and 104 days (O’Hara et al., 2020). To reduce the number of sick leave days, it is crucial to recognize the factors that affect this. Several factors have been associated with longer absence from work. These include level of education, blue-collar work, injury severity and receipt of compensation (Clay et al., 2010). Several characteristics associated with earlier return to work after injury have also been reported. These include a younger age, higher education, higher income, the presence of strong social support and employment in a white-collar job that is not physically demanding (Mackenzie et al., 1998). For DRF, pain and disability have been identified as the best predictors of the duration of sick leave (Egund et al., 2020).

The aim of this study was to compare the number of sick leave days between operated and non-operated patients in a nation-wide Finnish cohort. We hypothesized that operatively treated patients would return to work earlier than patients treated non-operatively. Additionally, we aimed to analyse the annual trends of operative treatment, amount of part-time sick leave, costs of different treatments and the incidence of DRFs in the working age population.

## Methods

In Finland, sick leave allowance, which depends on the collective labour agreement, is paid 10 days after the occurrence of sickness or injury. Before then, sick leave is paid by the employer. The Social Insurance Institution of Finland (KELA) holds records of all individuals who have received social benefits from the state, including sick leave allowance. However, if an injury is sustained from an accident at work, on the way to work or in a traffic accident, sick leave allowance is paid by a private insurance company, and these records are not included in this study.

Finland has a universal sickness insurance system that ensures all employees receive financial compensation for the time they are unable to work owing to sickness or injury. This compensation is controlled by KELA. After receiving a doctor’s certificate of disability or sickness, KELA may grant sick leave compensation to the employee. In some cases, it is also possible to work part-time (40–60% of normal working time) for a maximum of 120 days and receive financial compensation for the time absent from work. This is a feasible option when working full-time is not possible and working part-time does not affect recovery from injury. Part-time sick leave was first introduced in 2007 and the requirements for this have remained the same since 2010. Sick leave periods that are more than 90 days apart from each other are considered as separate incidents.

The Finnish National Hospital Discharge Register (HILMO), which is maintained by the Finnish Institute of Health and Welfare, holds records of all surgical procedures carried out in Finland. The register has high coverage and accuracy (Huttunen et al., 2014; Mattila et al., 2008; Sund, 2012). In Finland, register-based data include a unique personal identification number, which enables linkage between various registers. By combining the KELA records with records from the HILMO register, we were able to identify all patients operated on for a DRF. We included patients with both the Nordic Classification of Surgical Procedures (NCSP) procedure codes NCJ62, NCJ64, NDJ62, NDJ64 and NCJ99 and the International Classification of Diseases, 10th version (ICD-10 diagnosis codes S52.5, S52.6, S52.7, S52.8 and S52.9 for DRF). We then combined this information with data from the KELA records. Those patients who received an allowance but were not recorded in the HILMO registry within 90 days of the first day of sick leave were considered as non-operatively treated. The annual number of operations were also analysed according to HILMO data. All patients were included in this study and there were no inclusion or exclusion criteria determined.

The primary outcome was the number of sick leave days. Secondary outcomes were the number of partial sick leave days and the cost of treatment. The cost of sick leave was evaluated using the mean cost of one sick leave day calculated by the Confederation of Finnish Industries, which was estimated to be €300 (UK£257; US$323) in 2009 (Kaukinen, 2009) and €350 (UK£299; US$376) in 2015. When calculating the cost of surgeries, we used the diagnosis-related group (DRG) price of Tampere University Hospital for DRF surgery in 2016. The estimated cost per surgery was €1669 (UK£1427; US$1795). The total costs for the operative treatment group included the costs of the operation and the costs of sick leave. The costs for non-operative treatment included only sick leave. Owing to a lack of data, the occupation of the patients was not considered in this study. We also analysed the annual incidence of DRF by comparing the number in the working age population with the number of sick leave periods (data extracted from Statistic Finland website (stat.fi) on 6 January 2023).

### Statistical analysis

Based on visual evaluation of the histograms, the data for the partial sick leave days was normally distributed, but the data for sick leave days was not. The difference in sick leave days between groups were presented as medians and the Mann–Whitney U test was used to calculate statistical significance. The difference between groups in partial sick leave days was calculated using Student’s *t*-test. Linear regression was used to estimate adjusted group difference in each outcome variable. The covariates used were age and gender. We used R version 4.0.3 (Foundation for Statistical Computing, Vienna, Austria) for the data construction and IBM SPSS Statistics (version 27, New York, NY, US) for the statistical analysis.

## Results

Between 2010 and 2019, 19,995 patients in Finland had been on sick leave because of DRFs. We identified 20,650 periods and 1,344,615 days of sick leave during the study period. There was substantial variation in the number of sick leave periods between the years of the study. For example, there were 655 (36%) more sick leave periods in the year with the highest number of sick leave periods (2017) than in the year with the lowest (2014) (Table 1). However, no clear trend between the years of the study period and the number of sick leave periods was observed. A total of 625 (3%) patients with DRF had multiple periods of sick leave, indicating either multiple fractures or delayed complications after returning to work.

**Table 1.** The number of sick leave periods owing to attributable to distal radial fractures and the number of internal fixation operations for distal radial fracture annually between 2010 and 2019 in Finland.

Year	2010	2011	2012	2013	2014	2015	2016	2017	2018	2019	Total
Number of sick leave periods	1970	1979	1946	2218	1821	1913	2237	2476	2252	1838	20,650
Number of operations	291	365	322	455	404	388	496	557	574	494	4346
Percentage of patients operated	15%	18%	17%	21%	22%	20%	22%	22%	25%	27%	21%

During the study period, the annual number of operations increased from 291 to 494; the percentage of patients operated on increased from 15% to 27% (Table 1). A total of 211 patients were operated more than once. According to our data, the annual incidence of DRF in the working age population ranged from 6.8 to 9.3, with a mean of 7.7 per 10,000 population.

There were 4346 (22%) DRF patients in the operated group and 15,649 (78%) in the non-operated group. No major differences in age and sex distribution between groups were found (Table 2). In total, 21% of females and 23% of males were treated by operation.

**Table 2.** The characteristics of patients in each study group.

Group	Female	Male	Total	Median age (IQR)
Operated	3131 (72%)	1215 (28%)	4346	52 (42 to 58)
Non-operated	11,469 (73%)	4180 (27%)	15,649	52 (39 to 58)
Total	14,600	5395	19,995	52 (40 to 58)

To assess the impact of the surgery itself, we excluded the time the patient spent waiting for the surgery from further analysis. The mean number of sick leave days before the operation was 8.7 and 75.4 after the operation. In the operated group, the mean number of sick leave days was 75.4 and 62.6 in the non-operated group. The median number of sick leave days was 56 (IQR 42 to 79) and 43 (IQR 35 to 61) in the non-operated group (*p < *0.000). The number of sick leave days was 20% higher in the operated group. Based on a multivariable linear regression model, the age and sex adjusted difference was β 12.2 days (95% CI 10.0 to 14.3) higher in the operated group than in the non-operated group.

Most (71%) of the periods of sick leave lasted between 30 and 90 days in both groups (Table 3).

**Table 3.** The number of periods of sick leave lasting for 30 days or less, 90 days or less, 180 days or less, 365 days or less and more than 365 days.

Group	≤30	30–90	90–180	180–365	>365	Total
Operated	233	3074	709	296	34	4346
	5%	71%	16%	7%	1%	100%
Non-operated	2415	11,166	1293	662	113	15,649
	15%	71%	8%	4%	1%	100%

**Figure 1.**
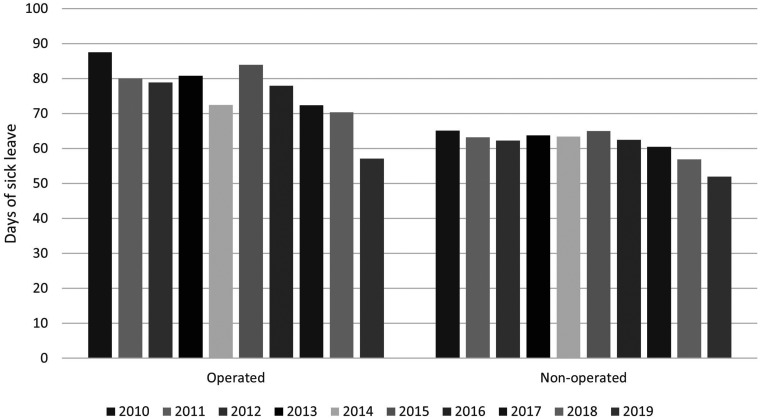
Mean annual number of sick leave days owing to distal radial fracture in Finland between 2010 and 2019.

The mean number of days of sick leave was 65.6 days for women and 64.6 days for men (95% CI –3.0 to 0.9). The mean number of days of sick leave fell from 60 days in 2010 to 47 days in 2019 for the operative group and from 45.5 to 41 for the non-operative group (Figure 1).

During the study period, 549 (3%) patients were on partial sick leave. Of these, 169 (3.9%) patients were in the operated group and 380 (2.4%) in the non-operated group. The mean number of partial days of sick leave was 57.0 and 63.9 days (95% CI –14.3 to 0.6) for the operated and non-operated groups, respectively.

The estimated overall cost of all sick leave days during the study period was €471 million (UK£403 million; US$506 million). The cost of sick leave per patient in the operated group was €29,411 (UK£25,155; US$31,627) and €21,905 (UK£18,735; US$23,555) per patient in the non-operated group. The overall cost of treatment in the operated group was €31,266 (UK£26,741; US$33,621) per patient. Therefore, the cost of the operative treatment of DRFs was €7505 (UK£6419; US$8070) and 34% higher than the cost of non-operative treatment. Furthermore, with the added cost of surgery, the difference between operative and non-operative treatment was €9361 (UK£8003; US$10,066) and thus the costs were 43% higher in the operated group.

## Discussion

In this study, we found that operated patients had 13 days more sick leave than those of non-operated patients. Partial sick leave was uncommon. Additionally, we found, that most of the costs associated with injury in both groups were largely owing to sick leave, with operative treatment itself only accounting for 5% of the overall treatment costs.

Our results are not in line with the findings of earlier publications reporting the functional results of different treatment options for DRF. Several recent meta-analyses have shown that operative treatment may result in better functional outcomes in working age patients at 3 months when compared with non-operative treatment (Linnanmäki et al., 2023; Ochen et al., 2020; Oldrini et al., 2022). It is often argued that the lack of long-term benefits should be considered when choosing between operative and non-operative treatment of DRF. However, improved functional outcomes at an earlier stage might result in an earlier return to work, as most patients returned to work within 3 months. On a national as well as on global level, an earlier return to work would result in a major reduction in costs. Hence, we should not overlook the importance of the improved functional outcomes of operative treatment in the working age population. In a retrospective analysis Toemen et al. (2021) showed that late mobilization patients had more than double the number of sick leave days (79 days) compared with early mobilization patients (34 days). It has also been shown that early mobilization after anterior locking plate operation can result in better functional outcomes and lower pain scores compared with late mobilization (Dennison et al., 2020; Quadlbauer et al., 2017; Watson et al., 2018). The use of partial sick leave was not common in our study. This was in line with the national register data (KELA): in 2021, only 10% of employees on sick leave used partial sick leave and only 6.7% of these were due to injuries. The use of partial sick leave could, however, enable earlier return to work without compromising the recovery process.

DRF is a common injury that causes significant costs to healthcare systems annually. Furthermore, there is some evidence that the incidence of DRF is increasing, particularly in the working age population (Jerrhag et al., 2017). Anterior plate fixation has been shown to be a cost-effective way to treat DRF when all costs are considered (Mulders et al., 2020). In the present study, the overall costs of treatment associated with DRF amounted to €0.48 billion (UK£0.4 billion; US$0.5 billion). However, the costs of operative treatment made up only a fraction of the overall costs. Swart et al. (2017) showed that the indirect costs of DRFs were a major contributor to the costs of overall treatment. Indirect costs accounted for 28% of the total cost for the operative group and 36% for the non-operative group. Absence from work was the major contributor to overall indirect costs, accounting for 93% of all indirect costs. Therefore, to reduce costs, the focus should be on regaining the functionality of the injured extremity and the fast return to work.

To reduce the number of sick leave days, multiple factors must be considered. The occupation of the patient is one major factor. Additionally, we should consider whether the injury is to the dominant or non-dominant hand of the worker, whether there is a possibility to modify the workplace conditions or work tasks or even provide additional training instead of regular work. The possibility of part-time work should also be considered. There is large variation in the duration of sick leave among patients with the same diagnosis, and the practices of prescribing sick leave have been shown to vary among physicians (Kankaanpää et al., 2013). With the implementation of national guidelines and improved education of physicians, we might be able to reduce the number of sick leave days.

In this study, we did not include the costs of the initial visits to hospital, radiography, follow-ups and immobilization. However, when these costs are included, the cost of operative treatment plays an even smaller role in total treatment costs. In recent years, there has been a steady increase in the number of operations involving the internal fixation of the radius. Moreover, according to data extracted from the National Hospital Discharge register, the number of such operations is still increasing. The overall number of operations performed as ‘Internal fixation of fracture of forearm using plate’ (NCSP, Nordic Classification of Surgical Procedures, code NCJ62) in Finland increased by 6.4% annually between 2012 and 2021 (data extracted from the Finnish National Hospital Discharge Register, HILMO on 6 January 2023). Operative treatment is associated with complications and these patients might be subject to additional operations, such as hardware removal, which can lead to additional sick leave days (Palola et al., 2021).

In the present study, we were able to collect accurate and reliable data from the Social Insurance Institution of Finland and the National Hospital Discharge Register (Huttunen et al., 2014; Mattila et al., 2008; Sund, 2012). These data include all patients who have received financial support from the state. However, the data do not include those patients who did not have sick leave at all or had sick leave that was not long enough to receive sick leave allowance from the state. These individuals are typically entrepreneurs and office workers.

There are several limitations to this study. The available data only included data from the Social Insurance Institution of Finland (KELA). Therefore, patients who sustained a DRF in work- and traffic-related accidents were not included. We were unable to consider the effect of the occupation of the patient on the number of days of sick leave. Our estimation is, however, that occupation is a major factor affecting the length of the sick leave period. We were also unable to collect data on the severity of the injury, the classification of the fracture or the indications for surgery. Thus, it is plausible that operated patients had sustained a more severe injury. We were also unable to identify patients, who were operated on outside Finland. It was not possible to identify those patients who had to undergo an operation after a period of conservative treatment. These patients were analysed as operated patients and only the number of sick leave days after surgery was used for further analyses. There were 252 patients who were operated more than 20 days after the first day of sick leave. Some of these patients may have undergone unsuccessful non-operative treatment. Patients with multiple injuries or other severe injuries might have had more days of sick leave, but we were unable to identify these patients.

In conclusion, we found that operative treatment did not reduce the number of sick leave days in this population.
